# Scrotal Paget’s disease: a case report and clinical analysis

**DOI:** 10.3389/fmed.2026.1780771

**Published:** 2026-04-09

**Authors:** Xuefeng Peng, Qiang Wang

**Affiliations:** 1 Guizhou Medical University, Guiyang, Guizhou, China; 2 Department of Urology, The Affiliated Hospital of Guizhou Medical University, Guiyang, Guizhou, China

**Keywords:** case report, pathology, postoperative follow-up, scrotal Paget’s disease, surgery

## Abstract

This study reports the case of a 67-year-old man with scrotal Paget’s disease. A systematic analysis of the clinical features, diagnostic and therapeutic strategies, and prognosis management of this rare disease was performed in conjunction with a literature review. The patient had a prolonged disease course with a history of three previous lesion excisions and presented this time due to lesion enlargement accompanied by mucous discharge. Dermoscopic examination revealed a “red background with punctate vessels,” and preoperative epidermal histopathology supported the diagnosis of Paget’s disease. The surgical approach involved wide excision of the lesion, aiming to remove the lesion as completely as possible while preserving the morphology and function of the surrounding tissues. Intraoperative frozen section analysis showed positive margins at the 1, 2, 6, and 11 o’clock positions. After further excision, immunohistochemical staining confirmed cytokeratin 7 (CK7) (+), gross cystic disease fluid protein-15 (GCDFP-15) (+), consistent with a sweat gland origin, and podoplanin (D2-40) (+), indicating a potential risk of lymphatic invasion. Finally, the patient was followed up regularly as planned, and at the most recent follow-up, no signs of recurrence were observed, and treatment outcomes were satisfactory, thereby allowing the discontinuation of further follow-up examinations. This case report provides a valuable practical reference for the standardized diagnosis and treatment of this rare condition.

## 1 Scrotal Paget’s disease

Scrotal Paget’s disease is a rare cutaneous malignancy, classified as a subtype of extramammary Paget’s disease (EMPD) (1). It predominantly affects elderly men aged 60–70 years, with a peak incidence at approximately 66 years. In a European study, the crude annual incidence of EMPD was reported to be approximately 7 cases per 10 million population; however, the true incidence remains uncertain (2). Clinically, scrotal Paget’s disease initially presents with pruritus, erythema, desquamation, or crusting of the scrotal skin, which may progress to erosion, ulceration, and serous exudation, with well-defined but irregular borders. It is often misdiagnosed as scrotal eczema, dermatitis, or tinea cruris. Definitive diagnosis relies on histopathological examination, revealing characteristic Paget cells—large atypical cells with prominent nucleoli, abundant cytoplasm, and eosinophilic staining—distributed in nests, cords, or islands within the basal or lower spinous layers of the epidermis (3).

We hereby report a clinical case of scrotal Paget’s disease diagnosed and treated at our center. The entire disease progression was comprehensively analyzed, with emphasis on the uniqueness of this case and its implications for clinical practice. The patient was an elderly man with a disease course of approximately 20 years. Initial symptoms included pruritus and discomfort, for which multiple treatment attempts had been made, including routine anti-inflammatory and anti-eczema therapies, with poor response and progressive enlargement of the skin lesions. The diagnosis was ultimately confirmed through pathological biopsy, and the patient underwent a wide excision of the lesion and flap formation surgery, with a margin of 1 cm beyond the positive lesion edge. During the subsequent 3-month follow-up, no recurrence or metastasis was observed.

As a rare disease, Paget’s disease of the scrotum is often unfamiliar to clinicians, leading to a high rate of misdiagnosis and a lack of standardized diagnostic and treatment guidelines. This case report aims to enhance clinical awareness of this condition, to optimize treatment strategies, and to improve patient prognosis, thereby providing valuable experience for clinical research on this rare disease.

## 2 Case presentation

### 2.1 Patient basic information and physical examination

During the data collection process for this case, strict adherence to medical ethical guidelines was maintained to ensure the patient’s privacy was fully protected. All data were anonymized and used solely for academic research purposes.

**Figure 1:**
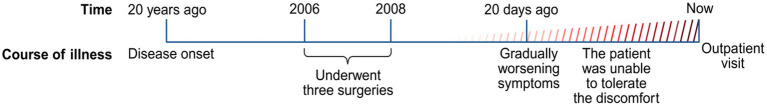
Patient’s clinical course timeline.

The patient is a 67-year-old man. He reports that, approximately 20 years ago, without obvious cause, he noticed erythema with a moist sensation and mucoid exudation in the scrotal, perineal, and right inguinal regions near the base of the penis. He sought medical attention at a hospital in Beijing, where the condition was diagnosed as Paget’s disease. Between 2006 and 2008, he underwent three local excision surgeries (specific procedures not detailed). He subjectively recovered well postoperatively. However, over time, he noticed that similar skin lesions recurred at the surgical sites. Twenty days ago, the patient noticed a worsening of the moist sensation and mucoid exudation at the scrotal lesion, accompanied by pruritus. For further treatment, he subsequently visited our hospital. To further illustrate the patient’s entire disease course, we have drawn a timeline of the disease process from the time of onset to the current visit (Figure 1).

Physical examination revealed a lesion on the surface of the patient’s right scrotum, approximately 3 cm x 5 cm in size. The affected skin area was thickened, with clear boundaries, and exhibited erythema, erosion, and exudation. A hard nodule was palpable, accompanied by mild pruritus and intermittent stabbing pain. It should be noted that, because the lesion involved the patient’s private area, the patient experienced shame and a strong avoidance tendency during the diagnosis and treatment; consequently, no preoperative clinical photographs were obtained.

### 2.2 Past medical history

The patient had a generally good health status, with no history of major chronic diseases such as hypertension, diabetes, or cardiovascular conditions. He denied smoking, alcohol consumption, or other unhealthy lifestyle habits, and there was no family history of similar skin disorders or genetic diseases. Prior to admission, he had not received any systemic or toxic drug treatments, thereby ruling out the influence of medication side effects or external factors on the current condition.

## 3 Course of diagnosis and treatment

### 3.1 Preliminary diagnosis

The preliminary diagnosis of scrotal Paget’s disease usually relies on the patient’s clinical manifestations, physical signs, and initial imaging and laboratory examination results. In this case, the patient primarily presented with symptoms such as local erythema, erosion, and pruritus of the scrotal skin. These symptoms closely resemble those of common dermatological conditions, such as eczema, which can easily lead to misdiagnosis.

**Figure 2:**
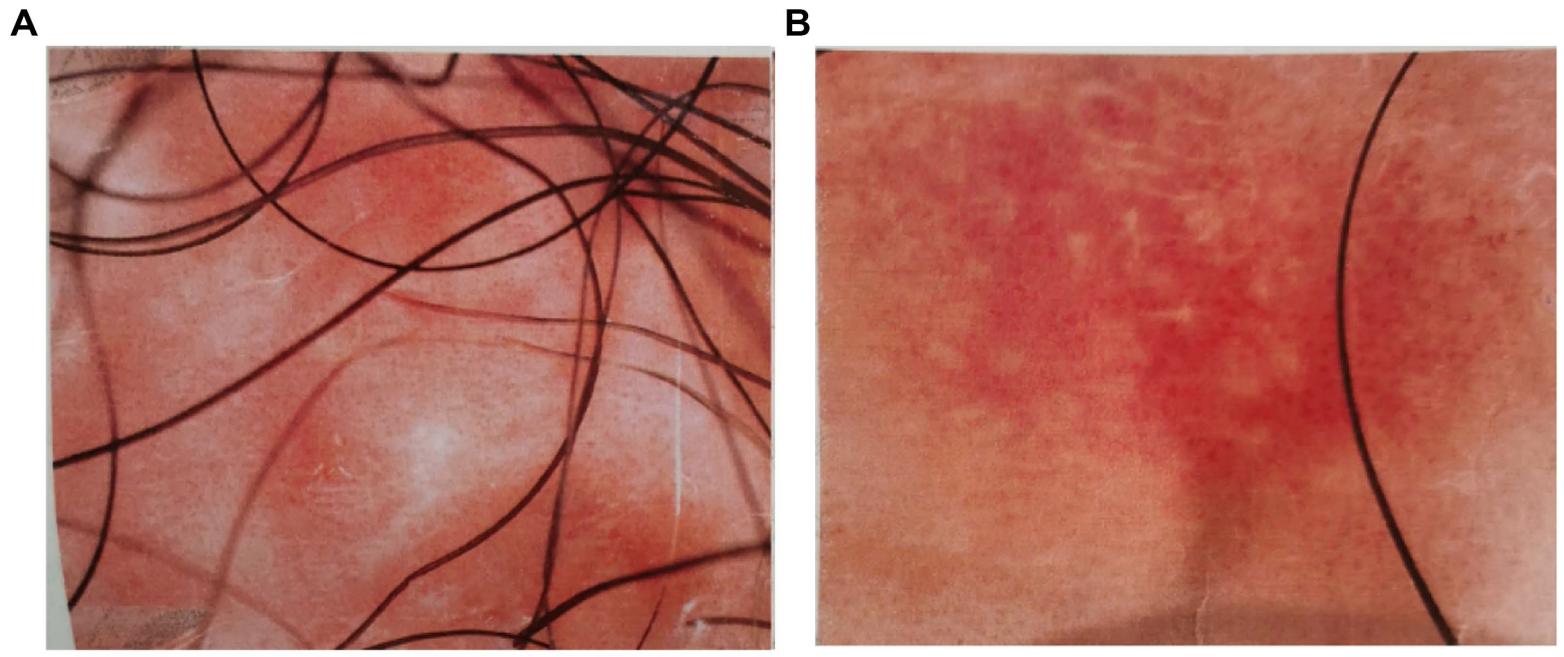
**(A,B)** Dermoscopic images of the patient’s lesion.

The dermoscopic examination conducted preoperatively revealed the following: “Under the microscope, the lesion area showed a red background with clear boundaries. Densely distributed, dotted, and globular vascular structures were observed in the center, with some areas ulcerated and accompanied by yellowish exudate. Scattered white fibrotic structures were seen in the center of the lesion, with a small amount of grayish-white scales attached to the surface” (Figures 2A,B).

**Figure 3:**
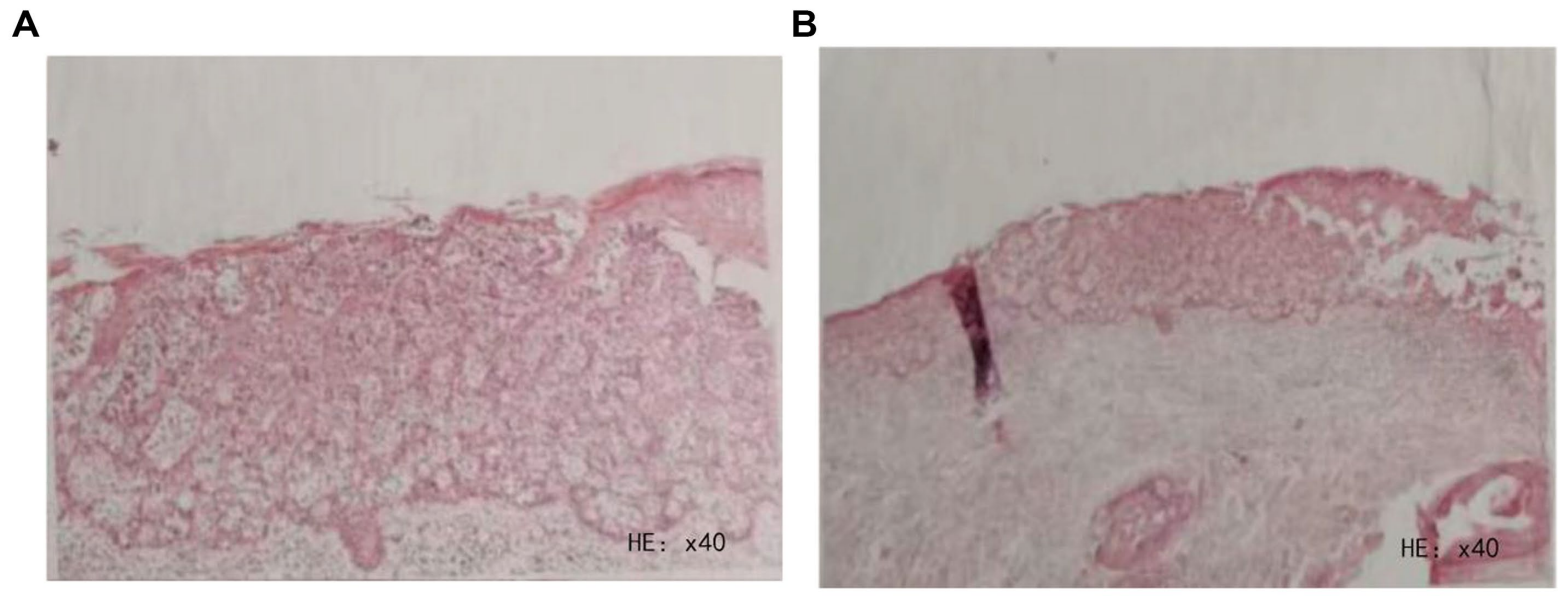
**(A,B)** Histopathological image of the lesional epidermal tissue.

It must be emphasized that the dermoscopic examination did not provide conclusive evidence regarding the nature of the skin lesion. Therefore, the patient underwent an additional epidermal tissue biopsy. The pathological findings supported the clinical diagnosis of scrotal Paget’s disease (Figures 3A,B).

Based on the above information, the patient was provisionally diagnosed with scrotal Paget’s disease.

### 3.2 Surgical procedure

Surgical excision is the cornerstone of treatment for scrotal Paget’s disease, and its success is directly correlated with patient prognosis. In this case, an extended excision of the lesion was performed. This approach aims to remove all diseased tissue while preserving as much normal tissue and function as possible.

**Figure 4:**
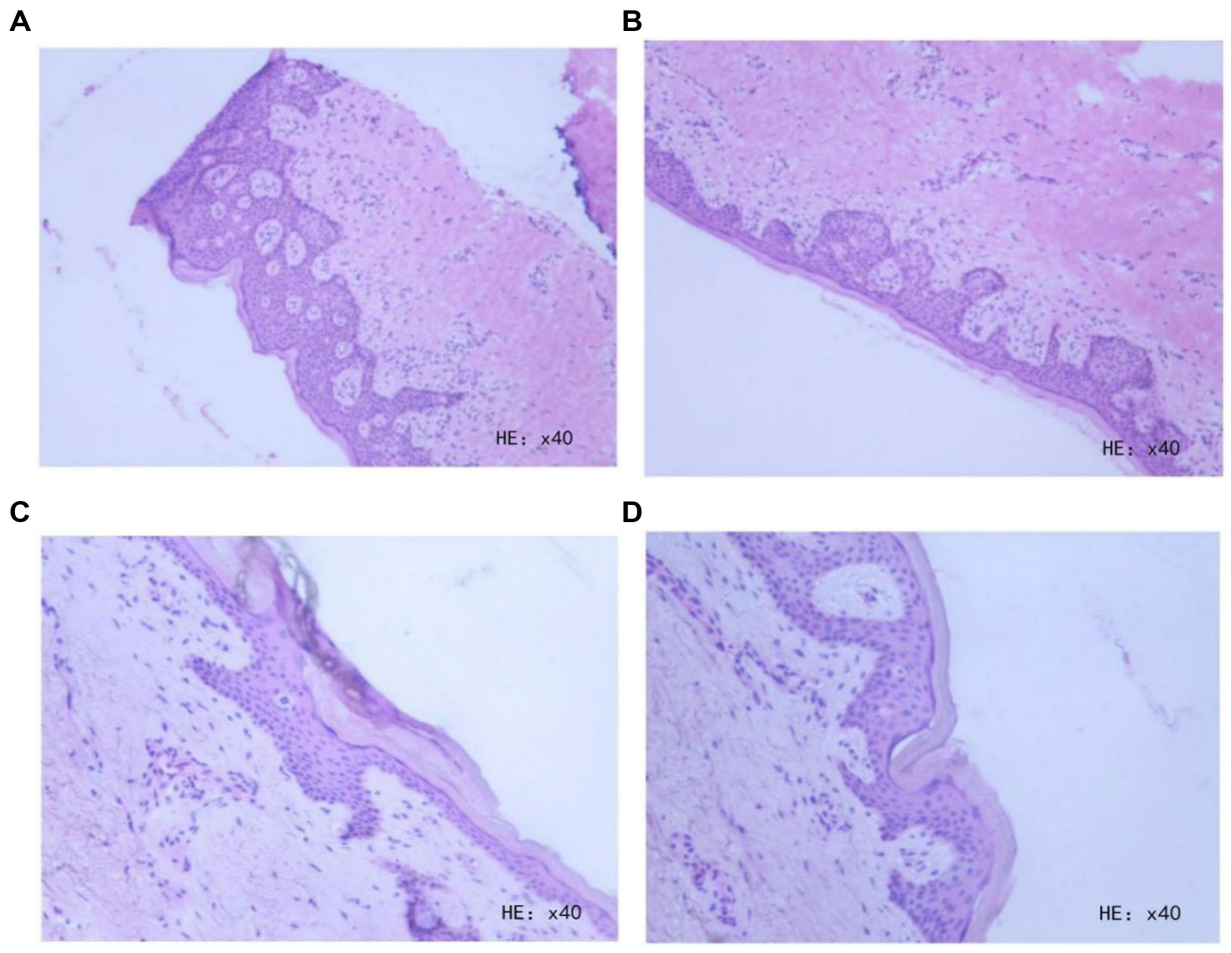
**(A)** Hematoxylin and eosin (H&E) staining of intraoperative rapid frozen section biopsy from the skin margin of the right inguinal region and scrotal lesion at 1–3 o’clock direction. **(B)** H&E staining of intraoperative rapid frozen section biopsy from the skin margin of the right inguinal region and scrotal lesion at 4–6 o’clock direction. **(C)** H&E staining of intraoperative rapid frozen section biopsy from the skin margin of the right inguinal region and scrotal lesion at 7–9 o’clock direction. **(D)** H&E staining of intraoperative rapid frozen section biopsy from the skin margin of the right inguinal region and scrotal lesion at 10–12 o’clock direction.

During the procedure, incisions were first made 1 cm beyond the lesion margins, cutting through the skin and subcutaneous tissue layer by layer. The superficial fascia was carefully dissected, and the tissues were excised. The surgical margins were labeled from 1 to 12 o’clock in a clockwise direction, and the skin edges were submitted for frozen section biopsy. The biopsy results indicated positive margins at the 1, 2, 6, and 11 o’clock positions, necessitating further extended excision (Figures 4A–D).

**Figure 5:**
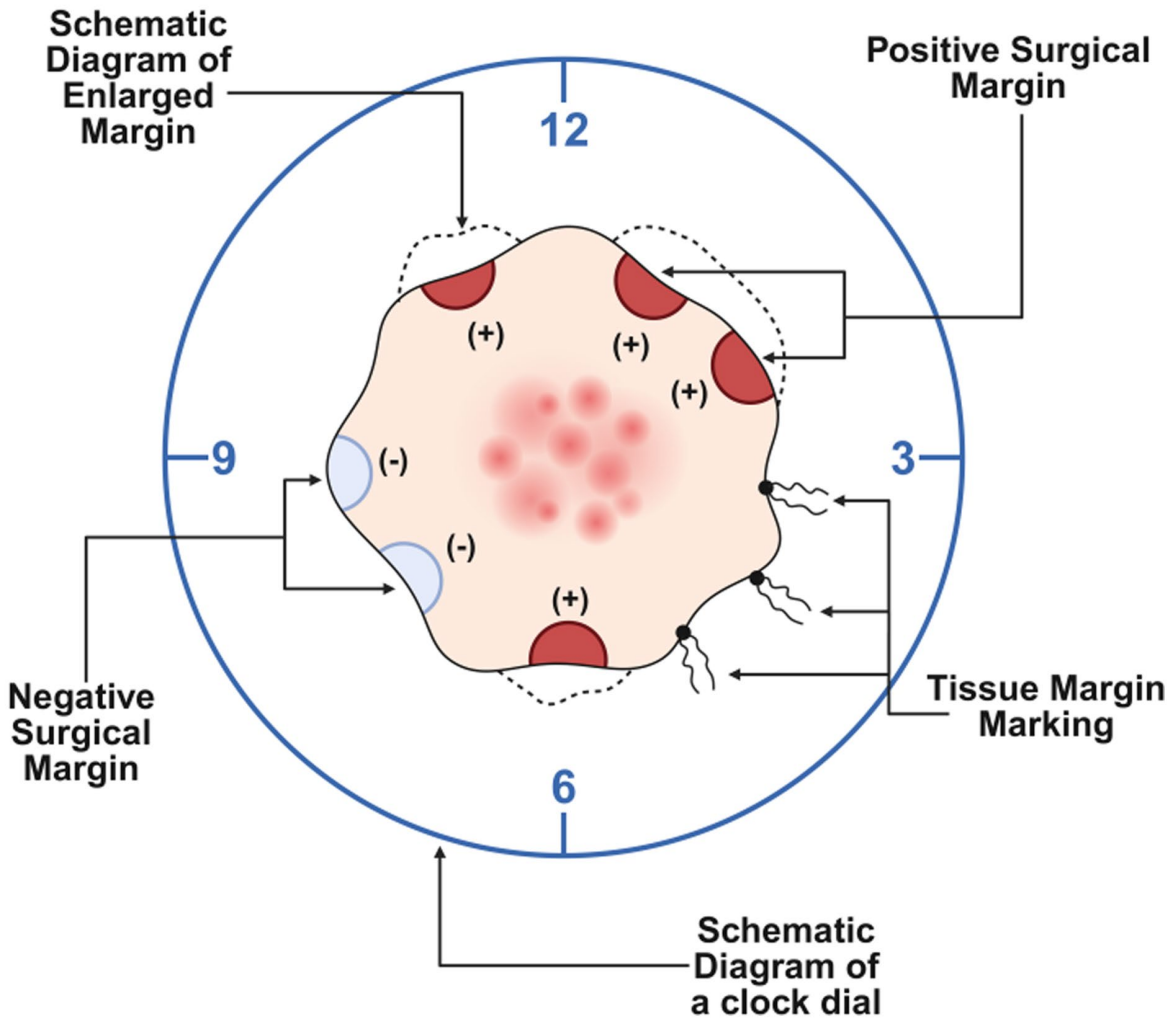
Schematic diagram of the margin control strategy.

Subsequently, we performed an additional 1 cm extension at the aforementioned sites. Routine pathological biopsies were performed on both the newly excised skin and the primary lesion. Finally, meticulous hemostasis was achieved, and the incisions were closed with layered, tension-reducing sutures. To illustrate the intraoperative margin control strategy, a schematic diagram describing the surgical process is provided (Figure 5).

### 3.3 Surgical outcomes

**Figure 6:**
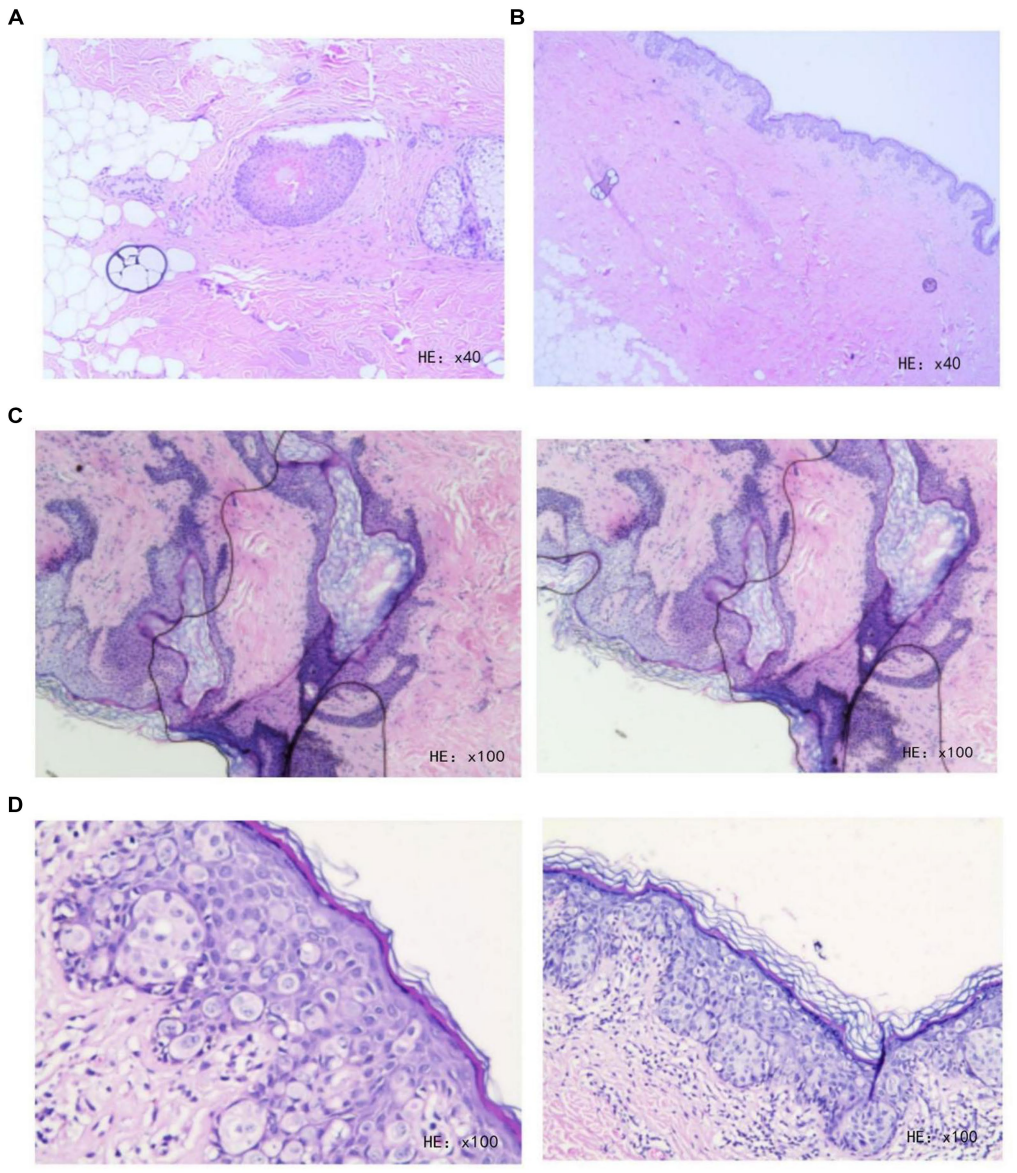
**(A)** H&E staining of frozen section biopsy from the skin margin of the right inguinal region and scrotal lesion at 1–3 o’clock direction. **(B)** H&E staining of frozen section biopsy from the skin margin of the right inguinal region and scrotal lesion at 4–6 o’clock direction. **(C)** H&E staining of frozen section biopsy from the skin margin of the right inguinal region and scrotal lesion at 7–12 o’clock direction. **(D)** Routine histopathological examination of the right inguinal region and scrotal lesion tissue.

The results of the routine postoperative pathology and the further pathological examination of the intraoperative frozen sections indicated that atypical cells were absent in the excised tissue from the extended margins. However, a few atypical cells were still observed in the epidermis of the skin tissue from the lesion center, consistent with Paget’s disease (Figures 6A–D).

#### 3.3.1 Postoperative immunohistochemical findings

**Figure 7:**
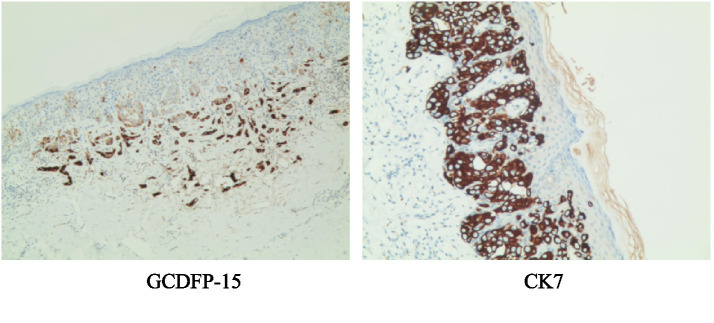
Immunohistochemical staining image of the right inguinal region and scrotal lesion tissue.

The findings of the postoperative immunohistochemical examination were as follows: CK7: positive (+), GCDFP-15: mostly positive (+), S100: positive in individual cells, synaptophysin (Syn): positive in individual cells, neuronal cell adhesion molecule (CD56): negative (−), chromogranin A (CgA): negative (−), D2-40: positive (+) in lymphatic endothelial cells, CK20: negative (−), and Cell proliferation-related protein (Ki-67): approximately 20% positive (+) (Figure 7 shows detailed immunohistochemical staining results).

#### 3.3.2 Postoperative pathological findings and immunohistochemical analysis

In the postoperative pathological examination, numerous typical vacuolated Paget cells were observed in the epidermal layer of the lesion tissue. These cells are characterized by large cell bodies, abundant pale cytoplasm, and large, deeply stained nuclei. The immunohistochemical results of the lesion showed CK7 (positive) and GCDFP-15 (positive) expression. Typically, the positive expression of CK7 and GCDFP-15 indicates that the tumor cells originate from the sweat glands or other glandular tissues, which is consistent with the typical origin of Paget cells (4).

Given the lesion’s proximity to critical endocrine glands, such as the testes, additional immunohistochemical markers were evaluated, including S100, synaptophysin (Syn), neural cell adhesion molecule (CD56), and chromogranin A (CgA), which are typically indicative of neuroendocrine origin (5–8). The results showed minimal positive expression in only a few cells, likely representing cutaneous nerve cell expression. Podoplanin (D2-40) was positive, suggesting possible lymphatic vessel invasion by the tumor (9).

The assessment of cytokeratin 20 (CK20) was primarily aimed at differentiating the lesion from other urological malignancies. Since the patient’s CK20 result was negative, other urogenital tumors were not considered (10). Ki-67 (20% positive) indicates active cellular proliferation within the lesion, consistent with the physiological behavior of malignant tumor cells (11).

The diagnosis of scrotal Paget’s disease was confirmed by postoperative pathology and immunohistochemical staining.

## 4 Postoperative recovery and follow-up

Although adequate margin management was performed during the surgery and no atypical cells were detected in the final pathology examination of the resection margins, the patient was advised to undergo regular follow-ups at defined postoperative stages due to the prolonged nature of the condition and the specific anatomical location. The patient consented to this follow-up plan.

However, because the surgery involved the patient’s private areas, he was unable to participate in follow-up procedures that required online video consultations or photography. This limitation was due to the sensitive location of the lesion and the patient’s feelings of embarrassment.

Therefore, we adopted a combined approach of telephone follow-ups and in-person consultations. Through verbal inquiries regarding the patient’s postoperative local recurrence, skin integrity, satisfaction, and quality-of-life improvements, we aimed to comprehensively evaluate the patient’s recovery status.

### 4.1 Follow-up results

Telephone follow-ups were planned to assess the patient’s recovery status at 1 week, 2 weeks, and 1 month postoperatively. At the 3-month time point, the patient was to be contacted by phone and advised to schedule a clinic visit for an in-person evaluation. Finally, a comprehensive prognostic assessment would be performed based on the data obtained during this in-person assessment.

#### 4.1.1 One week post-surgery

The scheduled follow-up 1 week after surgery was completed via telephone consultation. During this visit, we primarily focused on the early healing of the surgical site, screened for acute postoperative complications, and evaluated pain levels as well as limitations in daily activities. The specific assessment results are provided in Table 1.

**Table 1:** Follow-up status of the patient with scrotal Paget’s disease 1 week post-surgery.

Assessment dimension	Key indicators	Patient response	Prognostic assessment
Oncological assessment	Signs of local lesion recurrence	“I feel that the surgical site is not as itchy as it was preoperatively, and I have not felt any foreign bodies or lumps myself.”	No abnormal masses were palpated in the surgical area, suggesting non-enlarged lymph nodes and indicating a low short-term risk of lesion metastasis.
Surgical site recovery assessment	Dressing protocol	“I go for dressing changes biennially or triennially at the community hospital, with the last session taking place yesterday.”	The patient is undergoing regular dressing changes as scheduled.
	Healing status of the suture site	“I noticed some redness at the suture line when changing the dressing, but there is not much fluid drainage.”	Mild erythema and swelling in the surgical area are normal postoperative inflammatory responses; continued observation is recommended.
	Pain status	“I still feel some pain in the wound, especially when going up and down stairs, but I can still bear it.”	The patient’s pain score is below 5, indicating acceptable pain control.
Functional and quality of life recovery assessment	Activity limitation	“I can get out of bed and walk around independently, but I’m having a lot of trouble with dressing and using the toilet.”	Limited mobility in the early postoperative period is normal.
	Postoperative anxiety status	“This condition makes me feel very ashamed, and I’m also worried about the wound dehiscence and recurrence after surgery.”	The psychological anxiety is related to surgical trauma and the lesion in the private area; enhanced psychological counseling is required.
Postoperative complications and treatment adherence assessment	Postoperative complications	“I currently have no fever or other discomfort.”	The patient currently shows no signs of early postoperative complications and can continue with follow-up observation.
	Follow-up examinations and non-prescribed treatments	“I have not attended the follow-up appointment and has not received any other treatments.”	The patient has good compliance. The patient was advised to avoid and refuse unregulated/irregular treatments.

#### 4.1.2 Two weeks post-surgery

We also conducted the scheduled follow-up at 2 weeks postoperatively via telephone. Building on the findings from the 1-week follow-up, this assessment focused on the healing status of the surgical site, evaluated improvements in pain and daily activities, further assessed patient treatment compliance, and re-evaluated postoperative anxiety. The specific assessment results are provided in Table 2.

**Table 2:** Follow-up status of patients with scrotal Paget’s disease 2 weeks post-surgery.

Assessment dimension	Key indicators	Patient response	Prognostic assessment
Oncological assessment	Signs of local lesion recurrence	“I have not felt any lumps around my scrotum, and there are no similar rashes on the surrounding skin.”	There are currently no signs of lesion recurrence or local metastasis.
Surgical site recovery assessment	Healing status of the suture site	“When I was changing the dressing, I noticed that the surgical wound is not as red as it was last week, and there are no other abnormalities around it.”	The wound shows no redness, indicating controlled inflammation. With a significantly reduced pain score, the healing process is favorable. The patient may continue with regular dressing changes as prescribed until suture removal.
	Pain status	“I feel that the wound pain this week has improved a lot compared to last week.”	
	Dressing protocol	“I continued to change the wound dressing at the community hospital once every two days this week.”	
Functional and quality of life recovery assessment	Activity limitation	“I can carry out daily activities now, but I’m still worried about the wound getting contaminated when using the toilet. At the same time, I’m still worried about a recurrence.”	The patient’s ability to perform daily activities has improved, and psychological anxiety has been alleviated compared to before.
	Postoperative anxiety status	“As the wound is healing gradually, my sense of shame has been reduced significantly compared to last week.”	
Postoperative complications and treatment adherence assessment	Postoperative complications	“I have not seen any other abnormalities around the surgical incision myself. There are none of the conditions you asked about, such as necrosis.”	Based on the patient’s report, no postoperative complications are currently evident. With good compliance, the patient may continue with follow-up appointments.
	Follow-up examinations and non-prescribed treatments	“I have not received any other treatments.”	

#### 4.1.3 One month post-surgery

We also conducted the scheduled follow-up at 1 month postoperatively via telephone. Building on the findings from the 2-week follow-up, the focus of this assessment shifted from wound healing to the patient’s psychological recovery and restoration of daily activities. Furthermore, this follow-up included detailed instructions regarding the scheduled in-person visit at 3 months postoperatively and further assessed patient compliance. The specific assessment results are provided in Table 3.

**Table 3:** Follow-up status of patients with scrotal Paget’s disease 1 month post-surgery.

Assessment dimension	Key indicators	Patient response	Prognostic assessment
Oncological assessment	Signs of local lesion recurrence	“Just as before, I have not felt any lumps or foreign objects around the wound.”	Tumor control is favorable.
Surgical site recovery assessment	Healing status of the suture site	“I went to the community hospital last week to have the stitches removed, and I no longer need dressings to bandage the area. The area around my wound is not red now; there is just a pink line in the middle.”	Assessment indicates good wound healing; the description of “pink lines” is considered indicative of scar tissue formation, and the patient’s reported pruritus is likely associated with this scarring process.
	Pain status	“I feel that the wound is completely painless now, although the red line in the middle occasionally itches.”	
Functional and quality of life recovery assessment	Activity limitation	“I am now able to move around completely normally.”	The patient’s physiological function is nearly normal, and their psychological status is stable. The absence of sexual activity is likely related to the patient’s advanced age and decreased libido.
	Postoperative anxiety status	“Although I occasionally worry about a recurrence, I no longer have that sense of shame I had before.”	
	Improvement in sexual function	“I am old, and I do not have a sex life now.”	
Postoperative complications and treatment adherence assessment	Postoperative complications	“I do not feel the pulling sensation you mentioned around the wound, nor are there any signs of discharge or redness and swelling.”	No significant complications are noted; the patient demonstrates good compliance.
	Follow-up examinations and non-prescribed treatments	“I have already scheduled a follow-up outpatient appointment, and I am not receiving any non-prescribed treatments now.”	

#### 4.1.4 Three months post-surgery

In this evaluation, we designed the patient’s follow-up plan based on a comprehensive synthesis of the results from the first three postoperative assessments. Taking into account the patient’s tumor recurrence risk, surgical site healing, daily life recovery, and any complications, we made individualized adjustments to the previously designed examination plan.

Physical examination revealed no palpable enlarged inguinal lymph nodes, and no new visible Paget’s disease-like lesions were observed in the surgical area. Combined with the postoperative pathological results, the likelihood of recurrence at this stage was considered extremely low, making dermoscopy temporarily unnecessary.

The patient’s surgical incision had healed well. Sutured with absorbable sutures and benefiting from optimal incision design and suturing techniques, the current scar tissue on the scrotal surface appeared only as a slightly pink area that blends well with the natural folds of the scrotal skin. Therefore, scar repair treatment or further surgical scar excision was not required at this time. However, because the lesion and surgical site involved the patient’s private area and due to the patient’s sense of shame, he declined our request to collect clinical photographs.

The patient is currently fully self-sufficient in daily life, and no postoperative complications have been found. The surgical area shows no obvious pain, indicating satisfactory functional recovery; therefore, rehabilitation training is not currently recommended.

In summary, considering the disease status, the patient’s personal economic factors, and other relevant factors, we concluded that the patient can temporarily forgo additional auxiliary examinations.

### 4.2 Follow-up summary

Currently, the patient has completed the entire follow-up process. A comprehensive evaluation was conducted based on the results of three telephone follow-ups and one in-person visit. The patient was successfully followed up at all four assessment stages, demonstrating good compliance with no instances of follow-up interruption.

Throughout the continuous monitoring period, the patient showed no obvious signs of recurrence, exhibited good wound healing, gradually resumed daily activities, and experienced no postoperative complications. These outcomes collectively indicate a favorable surgical outcome and significant efficacy in subsequent disease management.

We have summarized the content of these four follow-ups in Table 4.

**Table 4:** Summary of postoperative follow-up for scrotal Paget’s disease.

Timeline	Core characteristics	Key intervention measures
1 week postoperatively	The wound was initially in the inflammatory phase, with mild functional limitation and significant psychological anxiety.	Wound care and pain management: Reinforce wound care to prevent infection. For pain control, oral non-steroidal anti-inflammatory drugs (NSAIDs) may be taken as needed, supplemented by psychological counseling (e.g., explanation of the healing process).
2 weeks postoperatively	Following resolution of inflammation and initial healing, the patient’s mobility improved.	Activity and procedures: Provide guidance on activity restrictions, dressing changes, and suture removal.
1 month postoperatively	The wound eventually achieved primary intention healing with functional recovery, and the patient’s psychological status stabilized.	Scar and lifestyle assessment: Evaluate scar formation and advise the patient that ointments may be used, if necessary, to improve scarring. Discuss sexual activity.
3 months postoperatively	Tumor control remains favorable, the quality of life is nearly normal, and no further follow-up is required.	Reassurance: Reassure the patient to alleviate anxiety and encourage a return to normal daily life.

## 5 Discussion and summary

Scrotal Paget’s disease was initially reported by Crocker in 1889. It primarily occurs in elderly men and exhibits clinical manifestations that are highly similar to benign dermatological conditions such as scrotal eczema and dermatitis, leading to frequent misdiagnosis and subsequent treatment delays for patients (12). In recent years, with the advancement of molecular biology research, new insights have been gained regarding the pathogenesis, therapeutic strategies, and prognostic evaluation of scrotal Paget’s disease. However, its low incidence rate and limited research data continue to hinder the standardization of clinical practice (13).

Histopathologically, the diagnosis is based on the presence of characteristic Paget cells within the epidermis. These Paget cells are large, round cells with pale cytoplasm, large and irregular nuclei, and an absence of intercellular bridges. They may contain multiple nucleoli or giant nucleoli, often exhibiting filamentous division. These cells are typically arranged in a nested, cord-like, or island-like pattern. In the advanced stages of the disease, although the number of Paget cells increases, they generally do not infiltrate the dermis. Instead, they are often separated from the dermis by the basal cell layer, although inflammatory infiltration may be present within the dermis. In some tumors, Paget cells exhibiting a signet-ring morphology or glandular structures often indicate poor differentiation and an increased risk of metastasis and invasion (14).

Immunohistochemical staining plays a significant role in diagnosis. Paget cells typically express cytokeratin 7 (CK7) and carcinoembryonic antigen (CEA) and are positive for periodic acid–Schiff (PAS) reaction, whereas they are usually negative for S100 protein, which aids in the differential diagnosis from other skin disorders (15). Recent studies have suggested that the development of scrotal Paget’s disease may be associated with the dysregulation of the Forkhead Box A1 - Anterior Gradient 2 (FOXA1-AGR2) axis and the overexpression of human epidermal growth factor receptor 2 (HER2) (16, 17). Nevertheless, due to the extreme rarity and paucity of cases, research into the underlying pathogenic mechanisms of this disease remains limited.

In clinical practice, the diagnosis of scrotal Paget’s disease primarily relies on histopathological examination. However, a comprehensive assessment that integrates clinical manifestations and patient history is essential, together with a differential diagnosis from benign skin lesions such as scrotal eczema and rashes, which significantly complicates definitive diagnosis.

Initially, patients often present with non-specific symptoms—such as pruritus, rashes, erythema, erosion, pain, and exudation. Due to the anatomical location’s sensitivity, patients frequently feel embarrassed, leading to delayed medical consultation or self-treatment for what is perceived as a common dermatosis, thereby failing to recognize the severity of the condition (18). As the disease progresses, worsening symptoms—including extensive skin infection, exacerbated pain and exudation, or even ulceration and hemorrhage—necessitate patients to seek medical attention.

Surgical treatment remains the mainstay of management. Currently, wide local excision is the treatment of choice. For lesions with limited extent, alternative approaches such as laser therapy or microsurgical techniques may be considered to eradicate the lesion while minimizing tissue loss (19, 20). However, these modalities carry potential risks of inadequate lesion visualization and incomplete tumor cell removal.

In the present case, the decision for surgical intervention was based on the following considerations:

1. Lesion size: 3 cm × 5 cm (suitable for local excision).
2. Previous surgical history: Laser or photodynamic therapy might be insufficient for microscopic lesions in the adhered area.
3. Patient age: 67 years (adequate surgical tolerance).

Generally, the surgical margin should extend at least 2 cm beyond the clinically apparent tumor margin through the full thickness of the scrotal wall, and negative margins must be confirmed before proceeding with flap reconstruction (21, 22). If deep tissue invasion is present, orchidectomy with spermatic cord resection should be performed.

Regarding inguinal lymph nodes, enlargement on the affected side is often due to inflammation rather than metastasis. Therefore, routine prophylactic lymphadenectomy is not recommended (23). Inguinal lymph node dissection is indicated only if lymph node biopsy is positive, with concurrent resection of the ipsilateral testis and spermatic cord considered on a case-by-case basis.

Adjuvant radiotherapy and chemotherapy are currently used as supplementary treatments. Recent studies have demonstrated that, for patients with HER2 overexpression, targeted therapies (e.g., tislelizumab) combined with chemotherapy (paclitaxel + cisplatin) show high efficacy and manageable toxicity (24). However, detailed research regarding other therapeutic modalities within the last 5 years remains scarce. Furthermore, photodynamic therapy, previously recognized as a potential treatment, is rarely adopted in clinical practice due to concerns regarding suboptimal efficacy (25, 26).

It must be emphasized that primary EMPD is associated with a high postoperative recurrence rate, ranging from 20 to 60%. Therefore, risk assessment for recurrence constitutes the paramount focus in the follow-up of scrotal Paget’s disease. A study indicated that the lesion boundary, degree of exudation, and nodule size are high-risk factors for postoperative recurrence, which may be attributed to tumor infiltration and inadequate surgical resection. Moreover, even if an intraoperative frozen section biopsy yields negative results, the possibility of postoperative recurrence cannot be excluded due to the high false-negative rate.

Nevertheless, literature regarding the postoperative recurrence of EMPD remains scarce to date, and a systematic, comprehensive follow-up guideline for scrotal Paget’s disease has not yet been established globally (27). Consequently, early detection, early diagnosis, and early treatment remain the most critical strategies for managing this condition.

In summary, scrotal Paget’s disease is a relatively rare malignant skin tumor with an incompletely understood pathogenesis and atypical clinical features. Currently, histopathological biopsy is the most crucial diagnostic modality. Upon confirmation of diagnosis, for patients eligible for surgical intervention, wide local excision of the lesion should be performed without delay. Intraoperative rapid frozen pathological examination is essential to ensure negative surgical margins, which significantly improves patient survival rates and reduces the risk of postoperative recurrence. Active postoperative surveillance is also required to promptly identify recurrence and enable timely management.

In this case, we adhered to the aforementioned conventional therapeutic regimen. However, we implemented a structured, planned, long-term follow-up protocol, using an individualized follow-up form to analyze and document the patient’s postoperative status. Limited by the patient’s individual circumstances, long-term follow-up data are currently unavailable. Future studies aim to conduct more detailed postoperative follow-up assessments.

## Data Availability

The original contributions presented in the study are included in the article/supplementary material, further inquiries can be directed to the corresponding author.
